# Validity of Internet-Based Longitudinal Study Data: The Elephant in the Virtual Room

**DOI:** 10.2196/jmir.3530

**Published:** 2015-04-16

**Authors:** Carys A Pugh, Kim M Summers, B Mark C Bronsvoort, Ian G Handel, Dylan N Clements

**Affiliations:** ^1^The Roslin Institute and Royal (Dick) School of Veterinary StudiesUniversity of EdinburghEdinburghUnited Kingdom; ^2^Royal (Dick) School of Veterinary Studies and The Roslin InstituteUniversity of EdinburghEdinburghUnited Kingdom

**Keywords:** epidemiology, validation studies as topic, Internet, questionnaires, longitudinal studies, health, canine

## Abstract

**Background:**

Internet-based data collection relies on well-designed and validated questionnaires. The theory behind designing and validating questionnaires is well described, but few practical examples of how to approach validation are available in the literature.

**Objective:**

We aimed to validate data collected in an ongoing Internet-based longitudinal health study through direct visits to participants and recall of their health records. We demonstrate that despite extensive pre-planning, social desirability can still affect data in unexpected ways and that anticipation of poor quality data may be confounded by positive validation.

**Methods:**

Dogslife is a large-scale, Web-based longitudinal study of canine health, in which owners of Labrador Retrievers were recruited and questioned at regular intervals about the lifestyle and health of their dogs using an Internet-based questionnaire. The Dogslife questionnaire predominantly consists of closed-answer questions. In our work, two separate validation methodologies were used: (1) direct interviews with 43 participants during visits to their households and (2) comparison of owner-entered health reports with 139 historical health records.

**Results:**

Our results indicate that user-derived measures should not be regarded as a single category; instead, each measurement should be considered separately as each presents its own challenge to participants. We recommend trying to ascertain the extent of recall decay within a study and, if necessary, using this to guide data collection timepoints and analyses. Finally, we recommend that multiple methods of communication facilitate validation studies and aid cohort engagement.

**Conclusions:**

Our study highlighted how the theory underpinning online questionnaire design and validation translates into practical data issues when applied to Internet-based studies. Validation should be regarded as an extension of questionnaire design, and that validation work should commence as soon as sufficient data are available. We believe that validation is a crucial step and hope our suggested guidelines will help facilitate validation of other Internet-based cohort studies.

## Introduction

The Internet has opened up the field of epidemiology by facilitating public engagement and removing the expense of postal and face-to-face data collection and processing. This is particularly relevant for longitudinal studies, which were prohibitively expensive to undertake on a large-scale basis in earlier eras. A corollary of this cost reduction is the need to rely on data generated solely by participants. These data are driven by what investigators hope are well-designed and validated questionnaires.

Re-using questionnaires already known to meet these criteria would minimize effort on the part of investigators. Future meta-analyses would be simplified if free and open access were given to standard questionnaires for different exposures or conditions. Considerable effort has been invested in designing and validating questionnaires that assess exposures, for example those gathering information on diet [1], alcohol intake [2], and smoking [3], and outcomes such as pain [4] and depression [5]. However, for many studies an appropriately tested questionnaire may not exist.

As outlined by Thrusfield [6], questionnaire validity refers to whether the answers truthfully reflect the issues designed to be addressed by the questions. In this paper, we report our experiences of trying to apply the well-understood theory behind data validation (see for example [6,7]) to data collected from members of the UK public. We assess the quality of a newly designed Internet-based questionnaire and use examples to support a series of suggested guidelines for checking the validity of any Web-based longitudinal health study. We report how specific problems such as the timing of checking procedures, the challenges of using user-derived measurements, and the impact of recall decay can affect the quality of data collected. This paper highlights why validation should always be considered as the crucial, final step in questionnaire design.

## Methods

Dogslife is the first large scale, Web-based longitudinal study of canine health [8]. In this project, owners of Labrador Retrievers were recruited and questioned at regular intervals about the lifestyle and health of their dogs using an Internet-based questionnaire. In December 2014, the number of participants reached over 5000 owners. The Dogslife questionnaire predominantly consists of closed-answer questions detailed in Multimedia Appendix 1.

Validity of the collected data could be affected at three levels: owner ability to recall incidents of interest, owner understanding of the questionnaire, and owner data entry errors. Quantifying the validity of longitudinal data is challenging as temporal changes (such as a subject growing in height or weight) complicate the simple test-retest scenario. The need to understand and maximize the extent to which Dogslife data reflected the experience of the dogs underlay all future work on the project. Thus, validation of these data had to be undertaken before detailed analysis could begin.

In our work, two separate validation methodologies were used: (1) direct interviews with 43 participants during visits to their households and (2) comparison of owner-entered health reports with 139 historical health records. The details of the sampling methodology and validation process can be found in Multimedia Appendix 2. The results are presented as specific validation problems, with examples of the results obtained. The changes made to the questionnaire in reaction to the findings are described in Multimedia Appendix 1.

## Results

### Timing of Validation

#### Overview

The timing of validation depends on the nature of the question being asked. First, early validation assessments allow questionnaire alterations to be instigated as quickly as possible, for example, where closed-answers given may be ambiguous (Example 1). Second, it is important to minimize the delay between a contributor answering the questionnaire and their data being validated. Factors that change with time, such as morphometric measures in growing individuals, are best assessed *before* they have time to change (Example 2). Finally, in the absence of information regarding the rate at which events will be reported, such as for the development of illnesses after the onset of participation, both early and late validation of data may be necessary (Example 3). Early assessment allows obvious reliability issues to be highlighted and immediately corrected, but insufficient events may have occurred to validate all answers. Later assessment will allow time for health records to accrue information for detailed validation (Example 3).

#### Example 1: Validation of Household Classification

During the visits to participants, it became apparent that participants were unclear as to what age a household member would be regarded as a “child” (Table 1), and owners were inconsistent when deciding whether another pet was “a member of the family” (Table 2). Answer options were revised (extended) with the aim of reducing misclassification (Multimedia Appendix 1). Owners were asked where their dog slept at night and following validation, an explicit option allowing contributors to say that their dog slept with another pet was introduced. After this amendment, the proportion of free-text answers to the sleeping location question dropped from 18.7% to 3.2% (χ^2^_1_=899, *P*<2.2x10^-16^).

**Table 1:** Comparison of household type in Dogslife record with visit response. Numbers entered refer to the count of households.

Dogslife	Visit response				
	Family, n	More than one adult, n	Retired, n	Single adult, n	Other, n
Family	13	3^a^	0	0	1^b^
More than one adult	0	14	0	0	0
Retired	0	0	6	0	0
Single adult	0	0	2^c^	4	0
Other	0	0	0	0	0

^a^Using “family” as a descriptor in the categories allowed owners to make their own judgment regarding what comprised a family. The intention had been to capture households including children under 16 years of age. Two of these visited households included children older than 16 years of age and the third was a married couple without children who regarded themselves as a “family”, rather than being “more than one adult”.

^b^This household comprised a couple who had children staying with them every weekend.

^c^Both households contained single, retired adults. Our categories were not mutually exclusive.

**Table 2:** Comparison of sleeping location in Dogslife record with visit response. Numbers entered refer to the count of households.

Dogslife	Visit response			
	Alone, n	Shared (family), n	Other (shared with dog)^a^, n	Other, n
Alone	24	1	2	0
Shared (family)	0	7	0	0
Other (shared with dog)^a^	0	0	8	0
Other	0	0	1	0

^a^Other (shared with dog) was not an option in the original questionnaire. During the validation, it became apparent that dogs that slept with other dogs were categorized inconsistently as (1) Alone, (2) Shared (family), and (3) Other: (with other dog). Introducing sharing with another pet as an explicit option prevents this inconsistency.

#### Example 2: Validation of Weight

**Figure 1:**
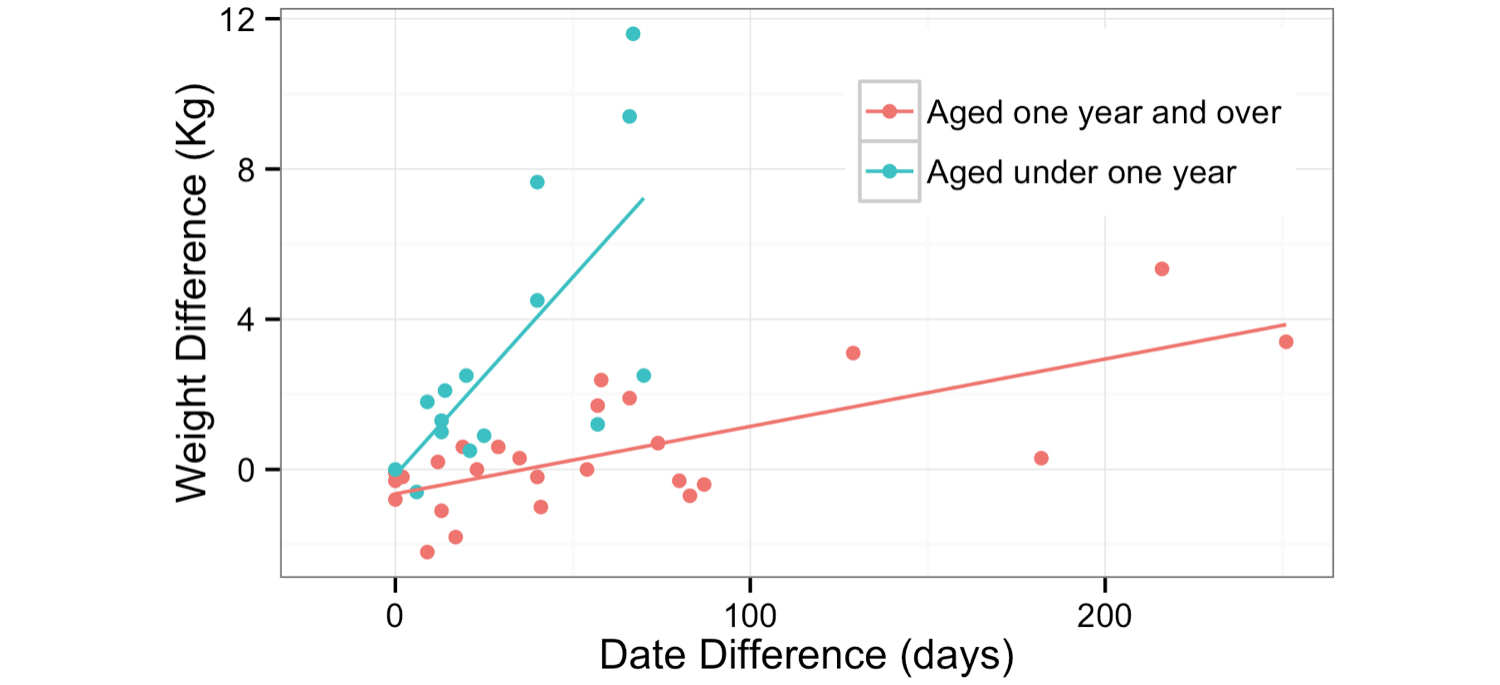
Weight misclassification compared to the time delay between online entry and validation visit (blue data points refer to dogs under 1 year of age and the red to those over 1 year of age, both with linear regression lines).

Weight was compared between the online record and the validation visits. The delay between participant contribution and subsequent validation, and the difference in weight measurements taken by the contributor and the investigator who took validating measurements are shown in Figure 1. The time that had elapsed between the owner completing the questionnaire online and the validation visit (where the investigator took the measurements) was typically too long.

#### Example 3: Validation of Health Records

Validation of owner-recorded health data by comparison to veterinary records was a two-stage process (Multimedia Appendix 2). Checking that the questionnaire addressed commonly occurring illnesses was possible at an early stage when little data had accumulated. However, a more quantitative assessment of recall decay and efficiency of reporting illnesses necessitated a delay to allow for sufficient events to have occurred.

### Data Not Validated

#### Overview

The lack of an external source for validation for certain data (eg, illnesses that do not present to a health care professional, or the use of drugs that can be obtained from multiple sources; see Example 4) highlights the potential value of such data. The information is unavailable elsewhere, but its value must be tempered by awareness that there is no robust way of assessing its accuracy.

#### Example 4: Validation of Over-the-Counter Medication

We tried to ascertain the accuracy of data collected about the timing of administration and products used for endo- and ectoparasitic control (worm and flea treatments). However, neither could be validated from veterinary records because these products can be purchased from multiple sources away from a veterinary surgery, and thus the veterinary record is not an accurate measure of their usage. Furthermore, during the participant visits, so few owners had these products at-hand that comparisons of brand with those entered in the record were impossible.

### Pitfalls and Unexpected Benefits of Validation

#### Overview

Although investigators may anticipate that certain data are more or less likely to be validated during the validation procedure, on the basis of their preconceptions and past experiences, such premonition may be misleading. The results of validation procedures revealed that the dietary information collected was representative for simple classifications, such as the type of food consumed by participating dogs. However, as was expected, it did not capture the full complexity of their diets (Example 5). Conversely, the expectation that other types of information might be challenging to collect accurately (such as dog weight) was confounded by the results (Example 6). Simply because one perceives a question to be difficult to answer should not preclude it from both the questionnaire and validation procedure.

#### Example 5: Validation of Diet

The Dogslife records and visit responses for simple classification of diet, such as whether it contained dried or a mixture of dried and tinned food, had good agreement (35/39 correct; see Table 3). Unfortunately agreement was poorer with more complicated diets, such as dried food with varying home-prepared additions. The reported food weight measurements were comparatively reliable for wet food but were of varying quality for the other food types where the weight could not be read from the side of a packet, indicating that owners were not weighing the food themselves. In some cases, the owner had documented the weight of a single meal, rather than the whole daily quantity of food (specifically requested in the questionnaire). Validation was possible but the process highlighted that detailed diet was not well captured.

**Table 3:** Comparison of food types in Dogslife record with visit response. Numbers entered refer to the count of households.

Dogslife	Visit response				
	Dried, n	Tinned, n	Dried & tinned, n	Home prepared, n	Other, n
Dried	27	0	0	1^a^	3^a^
Tinned	0	0	0	0	0
Dried & tinned	0	0	7	0	0
Home prepared	0	0	0	1	0
Other	0	0	0	0	4

^a^Four owners described their dog’s diets as “Dried” online but elaborated in person to describe a diet of dried proprietary dog food, with the addition of meat, vegetables, rice, gravy, and fruit.

#### Example 6: Validation of Weight Measures

**Figure 2:**
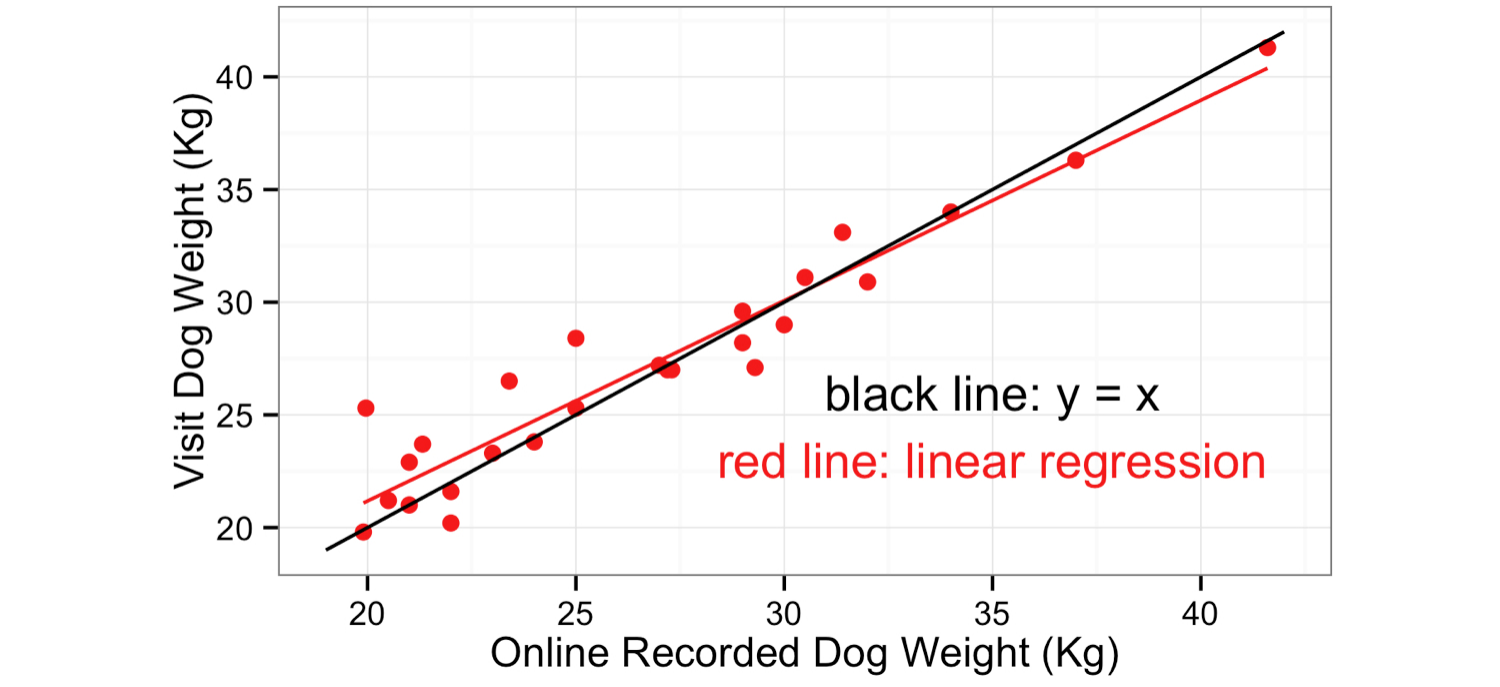
Comparison of online reported dog weight and visit measured dog weight for dogs over 1 year of age.

A comparison of online dog weight measurements for dogs over 1 year and the weights measured by the investigator during visits for the dogs over 1 year is shown in Figure 2. Lin’s concordance correlation coefficient showed moderate to substantial agreement between the two measures at .95 (95% CI 0.89-0.98) [9,10]. During the design stage of the questionnaire, it was decided that dog weight would not be a mandatory question because of perceived difficulty of measurement. Not only did validation indicate that weights given were representative, but 84% (95% CI 83.1-84.2) of data entries included a dog weight and many owners mentioned making special trips to the vet in order to have their dog weighed for the project.

### Social Desirability

#### Pitfalls and Unexpected Benefits of Validation

Subtle perception and societal pressures may have promoted misclassification for some questions either in the online questionnaire or during the validation visits. For example, the smoking status of household members (a seemingly simple question) was underestimated in the online questionnaire (Example 7), and this may have been due to social perception that smoking is undesirable.

#### Example 7: Validation of Smoking Status

Smoking status is addressed during the registration phase of the Dogslife study and was validated during owner visits (Table 4). The overall kappa score for agreement of smoking status was a moderate at .48 (SE=0.19), and there was a significant degree of misclassification among the low number of smokers. During visits, evidence of cigarette smoking was observed in the houses of people who had originally described themselves as non-smokers. With prompting, they said that they only smoked outside. This type of misclassification may have been due to the phrasing of the question: “Does anybody in the household smoke?”, which was perhaps interpreted as “Does anybody smoke within the household/house?”, thus classifying those who only smoke outside as non-smokers.

**Table 4:** Comparison of smoking in Dogslife record with visit response. Numbers entered refer to the count of households.

Dogslife	Visit response	
	Smoking, n	Non-smoking, n
Smoking	3	1
Non-smoking	4	33

### Recall Decay

#### Overview

**Figure 3:**
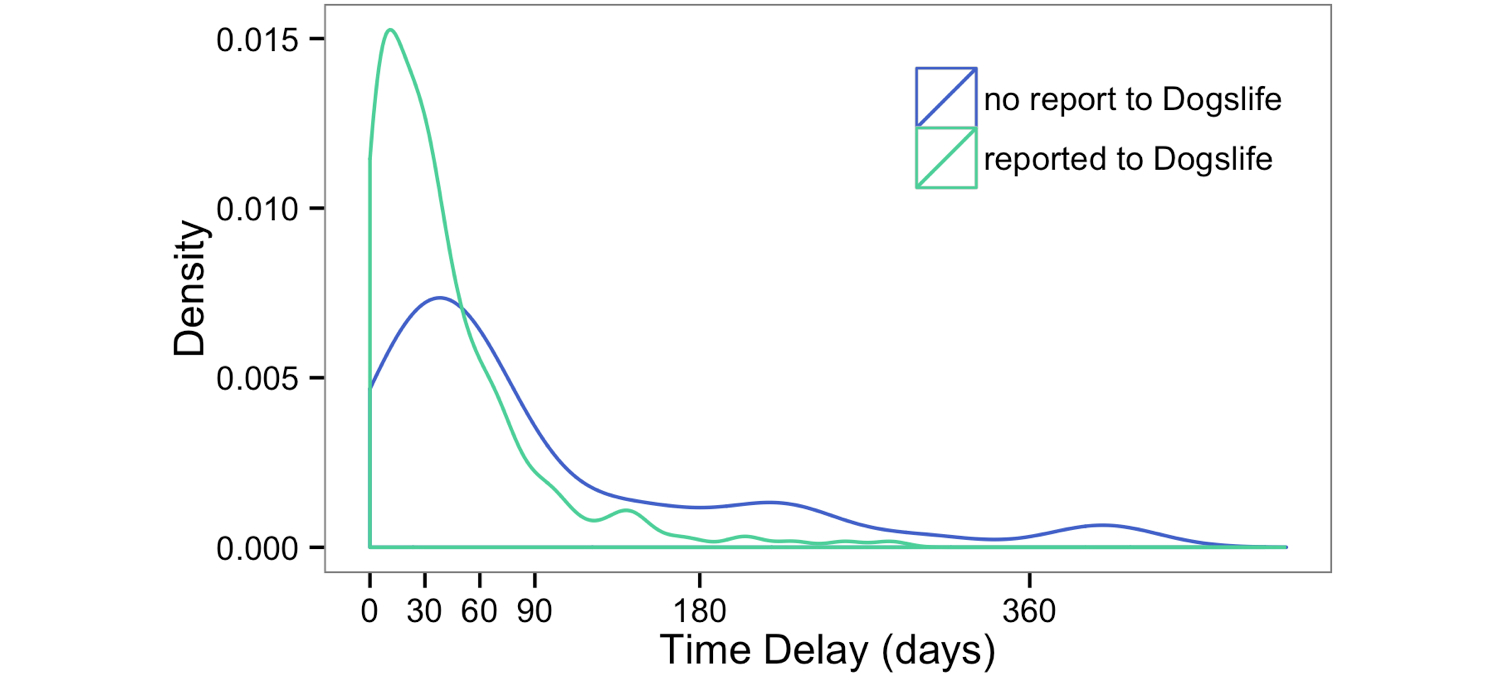
Vaccination recall decay plot (293 vaccinations, 127 dogs). Those not reported to Dogslife were vaccinations found in the veterinary record but omitted from the Dogslife questionnaire answers.

**Figure 4:**
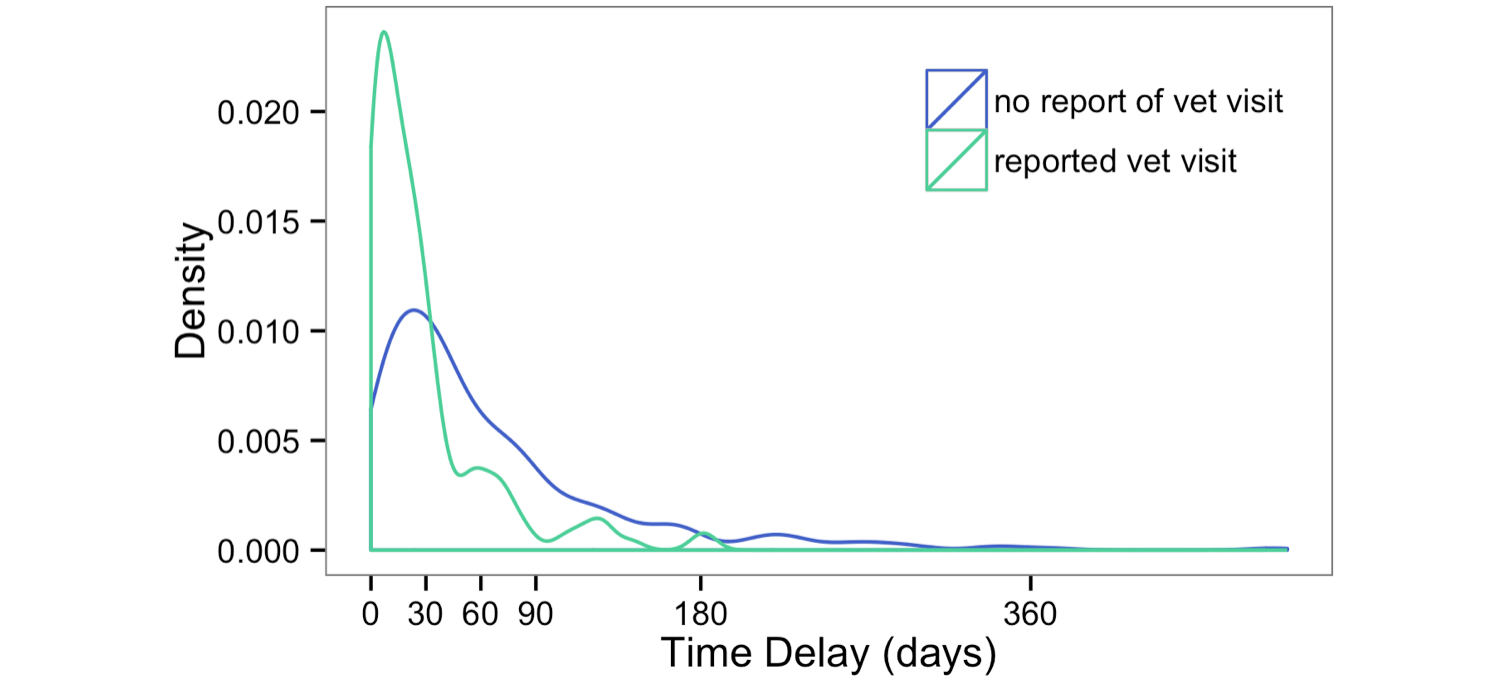
Illness recall decay plot (493 veterinary visits, 101 dogs). Those not reported to Dogslife were illnesses found in the veterinary record but omitted from the Dogslife questionnaire answers (22%).

We observed recall decay to differing degrees across the dataset through our validation procedure (Example 8), both with vaccinations (Figure 3) and illness reporting (Figure 4).

#### Example 8: Recall Decay of Vaccinations and Illness Reporting

A clear inverse association of the delay between vaccination and subsequent Dogslife data entry, and likelihood of reporting a vaccination was noted. Questionnaires that incorrectly omitted vaccinations were associated with a greater time delay (Figure 3). Participants may answer the questionnaire at any time, but recent analysis of the cohort return intervals shows a largely bimodal distribution with peaks at 37 days for owners of dogs under 1 year and 90 days, which is the requested time for 3-monthly data entries for dogs aged over 1 year. As such, the delay between an incident and the subsequent data entry (triggered by the reminder email) introduces the potential for recall decay.

Reporting of illnesses also appeared to be affected by recall decay. Figure 4 shows the delay between veterinary visit and subsequent Dogslife data entry. Illnesses that went unreported were again associated with a longer delay. The illness and vaccination plots have a remarkably similar shape indicating a similar pattern of recall among the owners for both questions.

### Method of Communication

#### Overview

Telephone conversations were more likely to elicit a positive response to the request for data than electronic communications (Example 9). It was not determined whether this was because those who permitted telephone contact were more likely to respond per se or whether the personal interaction involved in a phone call led to a better response.

#### Example 9: Automated Communication Does Not Eliminate Need for Personal Interactions

**Figure 5:**
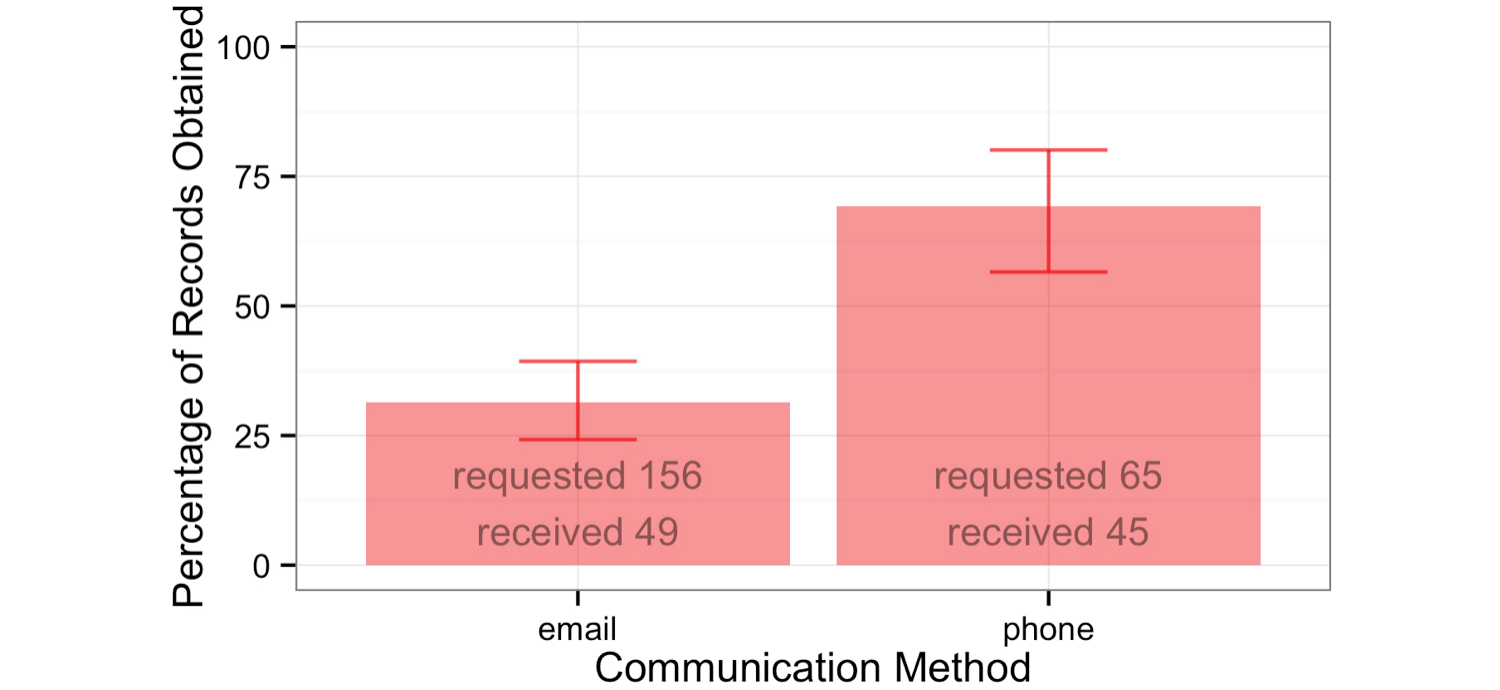
Health records obtained using telephone and email.

Owners were contacted and then sent a form to sign to request their dogs’ veterinary records. A greater proportion of records was obtained from owners contacted by phone than those contacted by email (Figure 5). A lower proportion of participants permitted phone contact (33% for telephone compared to 91% for email), which meant that email remained crucial, but the flexibility to use both methods of communication better facilitated engagement in the validation effort and project as a whole.

### Ease of Data Entry

#### Overview

Consistent feedback from owners of older dogs during the visit process was that their questionnaire answers largely did not change. They wanted to know why they kept being asked to fill in the same answers (Example 10).

#### Example 10: Pre-Population May Reduce Questionnaire Fatigue

In November 2013, sleeping location and dietary answers were pre-populated in the online questionnaire for dogs over 1 year of age (Multimedia Appendix 1). As a basic data check, answers that were not amended prompted a pop-up asking whether the owner was sure that nothing has changed. Given that the Dogslife project is based on the goodwill of owners, easing data entry was thought to be invaluable in retaining the cohort.

## Discussion

### Principal Findings

Now that we live in the era of big data, facilitated by the collection tools the Internet provides, the thorny question of validation is readily forgotten. Questionnaire design is an iterative process of design, re-wording, piloting, and re-wording again. Nevertheless, issues are still likely to arise when a finalized questionnaire goes live and “real” data are accumulated. As such, a questionnaire would ideally continue to be assessed with respect to collection of these data as a study progresses. With many longitudinal studies, recruitment is ongoing and immediate assessment of “real” data is impossible because too few entries are available to highlight problems. A balance therefore needs to be struck between the timing of any assessment, and the strength of the conclusions that can be drawn with potentially limited data. In a longitudinal context, it might be possible to explicitly treat the initial period of data collection as an extended pilot whereby the questionnaire could be refined to reflect issues raised during validity checks. Realistically, however, time and financial pressures make such a process unlikely; it is very hard to simply throw early data away. Starting validation efforts as early as possible means that issues may be addressed before too many data are collected.

In spite of extensive testing prior to launch [8], a number of the questionnaire answer options in the questionnaire were found to be problematic (Example 1). The options that were offered affected the validity of several measures. Contributors varied in their interpretation of the household descriptor “family”, and dogs that slept with another pet were inconsistently categorized as either “sleeping alone” or “sleeping with a member of the family”. By waiting to start our validation until we were ready to start analysis of our hypotheses (22 months after launch), we missed the opportunity to correct such simple issues with the questionnaire early in the project. In retrospect, we should have identified these issues by starting our face-to-face validation as soon as there were enough participants and data (in our study, this was approximately 3 months after launch). For longitudinal studies, a questionnaire must be quick and simple for the participant in order to maximize retention of the cohort. A balance was struck throughout the original questionnaire between clarity and brevity, and in the examples outlined above, brevity introduced potential errors into classifications.

Changes in height and weight in dogs aged under 1 year of age (ie, dogs that were still growing) made validation of answers to these questions challenging. Delays between questionnaires being answered and owner visits also had wider ramifications for the validation process. We wrongly believed that participants would be reluctant to take part in the visit-based validation exercise, and consequently a wide time interval between last online data entry and proposed visit was chosen to create a large sampling frame. With hindsight, it would have been preferable to select from those who have given data entries within a very short time. The interval can be widened if participation rates are low. A wide timeframe impacted our ability to make useful comparisons because of changes in the household situation since data entry.

Typically, for the avoidance of the issues described above, validation should be timed closely to data entry but with contemporaneously collected data such as health records, review can take place at any time. The event count for such types of data will increase as participants age, and consequently more information can be assimilated for every record collected if assessment is delayed.

Ideally, each parameter would be validated against a reliable external data source (eg, the records of clinic visits). For certain parameters, such as illnesses that do not result in a visit to health professionals, external sources may not exist. This was a facet of health behavior that we were unable to validate directly. Alternative techniques such as daily record keeping have been used in activity studies, but these methods themselves may not be robust [11].

Many studies based on online questionnaires report minimal or no validity checks, for example [12] and [13]. Time and money are limited in any project, and finding problems with data both delays analyses and makes them more complex. Some data, for example individual human dietary information, are notoriously difficult to collect, as they vary from day to day, both in type and quantity, and people have difficulty accurately recalling details [14]. Given the relatively monomorphic diets fed to pet dogs, we anticipated that dietary information would be accurately recorded by the questionnaire. However, keeping the questionnaire relatively brief was incompatible with the unexpected complexity of canine diets. The apparent difficulty owners had with weighing and reporting daily intake was at odds with what had been perceived to be a simple measuring process. By contrast, it was anticipated that owners might have difficulty weighing a relatively large breed dog and that reported dog weights might suffer from underestimation similar to those found in human studies [15,16]*.* The validation procedure confounded our expectations, highlighting its utility, but also demonstrating risks involved. If data are put up for validation, the investigators must be prepared for them to be shot down.

Social desirability may lead to inaccurate responses. Self-reporting of smoking status is thought to be reliable in the general population [17] but is affected by social factors in specific groups such as pregnant women [18] and those suffering from respiratory diseases [19]. We observed misclassification in response to a relatively simple question that may be attributable to the social perception of the undesirability of smoking.

Recall decay may affect any study where events are reported in retrospect and is thought to reflect the declining strength of memory [20]. It is taken into account in order to optimize response to censuses [21], specifically studied with regard to the accuracy of self-reported drug misuse [22], and must be considered in the design of all questionnaires. To try and circumvent the problem, investigators can permit participants to enter data at any point; something that online studies are ideally suited to. Unfortunately, this does not necessarily eliminate the problem (Example 8).

If recall decay remains an issue, one can focus on events reported during a number of days prior to each data entry (a small “at-risk” period) rather than that subject’s whole record. This minimizes misclassification of subjects as controls when they should be cases. Schmidt et al [23] used this technique in a longitudinal study of the prevalence of diarrhea, and while it reduced the quantity of available data, quality was improved. Unfortunately, precise quantification of the effect of recall decay (such as determined by [24]) cannot be made with a relatively small number of health records. Using a limited “at-risk” period and, for high prevalence diseases, requesting further records to ensure accuracy of case and non-case status, will more broadly increase the power of risk factor studies.

The design of Dogslife as an Internet-based project should have minimized recall decay as owners may enter new information at their convenience and were not limited to reporting when an interviewer visits or the next questionnaire comes through the post. However, the interval between Dogslife data entries peaked at 37 days for dogs under 1 year [8] and 90 days for dogs aged over a year. Owners were returning when they received reminder emails, not when there was a recent incident to report.

The utopic goal of Internet-based longitudinal studies is that all communication can be automated and electronic to minimize costs. However, in our experience, while email was the favored method of contact for participants, telephone conversations were more likely to elicit a positive response. It seems it may not be practical to move everything into the virtual realm just yet.

Bias from loss to follow-up is a chronic and well-recognized problem in longitudinal studies [25]. Investigators need to design their questionnaire to minimize fatigue in participants who will hopefully answer it time and time again. One key decision driven by feedback from our validation process was pre-population of the questionnaire (Example 10). Mooney et al [26] addressed the issue of pre-populating factual answer fields as part of their longitudinal study of substance abuse. They showed that offering partially pre-populated questionnaires resulted in minimal time saved for those who completed them but was perceived to be “very helpful”—a positive effect on perception rather than reality. There is obviously a balance to be struck between reducing data quality and minimizing retention bias. Online studies can build checks into any pre-populated questionnaire so as to hopefully minimize any negative impact of pre-population on data quality. Where some answers do not change greatly with time, pre-population may be the lesser evil.

### Conclusions

Testing the reliability and validity of longitudinal data captured by electronic means is crucial to substantiating any analyses based on those data. A plan for validation should be part of the design of a project. This builds quality into the science and ensures that funders do not expect immediate analyses. On the basis of our results, we suggest considering the following:

1. Timing is everything. Do not make validation the first stage of analysis; make it the last stage of questionnaire design. Initial validation should be performed as early as possible, particularly direct visitation as it may reveal or highlight unexpected issues not identified in pre-testing. It should also be appreciated that each question will have its own optimal time point for validation (both the time from the start of the study and the time between data entry and the validation procedure). When using contemporaneous health records, delaying allows time for data to accrue.
2. Some questions cannot be checked for reliability or validity. This should be recognized, accepted, and reported by the study.
3. If there is a delay between event occurrence and reporting, then expect recall decay. Try to determine how much impact it is having on the study. If participants have long intervals between contributions, then analyses may have to be changed to accommodate the “missing” timeframe.
4. Providing a non-automated alternative to Internet-based communication may aid in participation. This applies not only to validation efforts but also to potential detailed work with subsamples of a cohort.
5. Expect the unexpected. This is particularly relevant with regard to user-derived measurements taken by participants. Validation implies an assessment of the unknown. It can be disheartening but should not be regarded as simply a burden because it can have unexpectedly positive results*.* Prior concerns with regard to aspects of a questionnaire may be belied by positive validation results.

While validation has the potential to be a somewhat thankless task, our efforts generated positive feedback from our participants. The process also highlighted some issues that might have been missed if face-to-face visits had not been conducted. Our validation work not only means that we understand our data, but we believe it will help reduce future loss to follow-up and minimize selection bias.

## Multimedia Appendix 1

Elements of the Dogslife questionnaire with the original response options and the modified response options (in bold font) following the validation exercise (the Dogslife questionnaire is copyrighted to the University of Edinburgh).

## Multimedia Appendix 2

Validation methodology.
