# Supplementary material for: Validity of Internet-Based Longitudinal Study Data: The Elephant in the Virtual Room
Source: J Med Internet Res. 2015 Apr 16;17(4):e96. doi: 10.2196/jmir.3530 (PMC4416133; doi:10.2196/jmir.3530)
Supplement: Supplementary file 1 [file jmir_v17i4e96_app1.pdf]

Appendix 1: Elements of the Dogslife questionnaire with the original response options and the modified response options (in **bold** font) following the validation exercise. The Dogslife questionnaire is copyrighted to the University of Edinburgh.

Question<br>[Question title]	Original Response Options	Revisions/Comments
[Household]<br>How would you describe your household?[r]	- Family (one or more adults and one or more children)<br>- More than one Adult<br>- Single Adult<br>- Retired (Single or Couple)<br>- Other[a]	Revision of response options<br>**- One or more adults and one or more children under 16**<br>- More than one adult<br>- Single adult<br>**- One or more retired adults**<br>- Other[a]
[Smoking Status]<br>Does anybody in the household smoke?[r]	Yes/No	
[Dog Purpose]<br>Do you own your dog primarily as a:[r]	- Household pet<br>- Working dog (eg. Gundog)<br>- Assistance dog (eg. Guide Dog)<br>- Other[a]	
[Other Pets]<br>Are there other pets in the household?[r]	Yes/No	
If *Yes* to above<br>[Other Pets Types]<br>- What species and how many?[r]	- Dogs<br>- Cats<br>- Other[a]<br>(Each with a drop down list of numbers to choose from)	
[Sleeping Location]<br>Where does Dog* sleep at night?	- Alone in a room in house<br>- In a room shared with a member of the family<br>- Outside<br>- Other[a]	Pre-population and revision of response options<br><br>- Alone in a room in house<br>**- In a room shared with a person**<br>**- In a room shared with a pet**<br>**- In a room shared with a pet and a person**<br>- Outside<br>- Other[a]

[Feeding Frequency]<br>At present, how many times a day do you feed Dog*?	- Once daily<br>- Twice daily<br>- Three times daily<br>- More than three times daily	Pre-population and revision of response options<br><br>- Once daily<br>- Twice daily<br>- Three times daily<br>- More than three times daily<br>- **Throughout the day**
[Feeding Timings]<br>When do you feed Dog*?	- In the morning<br>- In the evening<br>- In the morning and evening<br>- Throughout the day<br>- Multiple times throughout the day	Pre-population and revision of response options<br><br>- In the morning<br>- In the evening<br>- In the morning and evening<br>- **In the morning, lunchtime and evening**<br>- Throughout the day<br>- Multiple times throughout the day
[Food Types]<br>What types of food do you give to Dog*?	- Dried food<br>- Tinned food<br>- A mixture of dried and tinned dog food<br>- Home prepared food<br>- Other[a]	Pre-population
If *dried*, *tinned* or *mixture* are chosen above (using dried as an exemplar)<br>[Food Brands]<br>Which dried foods do you feed Dog*?	19 different brands or supermarkets with tick boxes<br>- Other[a]	Pre-population
[Food Quantity]<br>How much dried food do you feed Dog* each day? Enter a number, either in grams or ounces.	Free text box to type number with adjacent drop down box to choose ounces or grams.	Pre-population
[Titbits]<br>Does Dog* also receive titbits or left-over food from your own meals and snacks?	Yes/No	Does Dog* also receive ``titbits''? **For example anything else your dog eats such as food off your plate, training treats, chews etc?**
[Bathing]<br>Has Dog* been bathed in the last 4 weeks?	Yes/No	Has Dog* been bathed **since you last visited Dogslife?**

If *yes* to above<br>[Bathing Frequency]<br>On average how often do you bathe Dog*?	- Daily<br>- Twice Weekly<br>- Weekly<br>- Fortnightly<br>- Monthly<br>- Less than monthly<br>- Never	
[Bathing Material]<br>What do you use to bathe Dog*?	- Water alone<br>- Dog shampoo<br>- Human shampoo<br>- Other	
[Dog Height]<br>How tall is Dog*?[i]	Drop-down list of numbers with adjacent drop-down choice of cms and inches	Height originally asked until dog one year of age. Changed so that it is now asked until 18 months of age.
[Dog Weight]<br>How much does Dog* weigh?[i]	Drop-down list of numbers with adjacent drop-down choice of kg and pounds	
[Vaccinations]<br>Has Dog* been vaccinated since you last visited the site?	Yes/No	
If *yes* to above<br>[Vaccination Timings]<br>When was Dog* vaccinated?	Drop-down calendar	
[Vaccinated Diseases]<br>Which diseases was Dog* vaccinated against?	- Routine Vaccinations (defined as distemper, canine hepatitits, parvovirus, leptospirosis)<br>- Kennel Cough<br>- Rabies	
[Wormings]<br>Has Dog* been wormed since you last visited the site?	Yes/No	
If *yes* to above<br>[Worming Timings]<br>When was Dog* last wormed?	Drop-down calendar	
[Wormer Brand]<br>What is the name of the product you use?	9 different brands with tick boxes<br>- Other[a]	
[Fleas]<br>Have you used any products to prevent or	Yes/No	

treat fleas since you last visited the site?		
If *yes* to above<br>[Fleas Type]<br>What type of flea control product do you use?	- Collar<br>- Oral Medication<br>- Drops or 'spot-on' for the dog's coat<br>- Spray for the dog's coat<br>- Spray for the house	
[Illness]<br>Has Dog* had any of the following problems since you last visited the site?	- Vomiting<br>- Diarrhoea<br>- Coughing<br>- Scratching<br>- Licking or chewing<br>- Limping or lameness<br>- Any other[a] illnesses or problems<br>Each with an adjacent choice between yes/no.	
[Illness Start]<br>If *yes* to any of the above (using vomiting as exemplar)<br>Approximately when did the vomiting start?	Drop-down calendar	
[Illness End]<br>Approximately when did the vomiting get better? If your dog is not better yet, click the box below.	Drop-down calendar<br>Tick box titled 'My dog is not better yet'	
[Illness Vet Visit]<br>Did you take Dog* to your vet for the vomiting?	Yes/No	
If *yes* to above<br>[Visit Vet Date]<br>Approximately when did you visit the vet?	Drop-down calendar	
		New question added if *no* regarding vet visit<br>**Do you know why Dog* developed this illness?**<br>Free-text box for response

[a] If 'other' is selected, a free-text box appears where the owner may specify their answer

[i] A question mark titled 'How do I measure my dog's height/weight' is visible next to the question. If selected a pop-up appears with a short video, a series of written instructions and a series of photographs showing the suggested measurement process.

[r] Questions asked as one-off when owner registers with project. Owners may change these answers by clicking on ``Edit your profile'' on the Dogslife homepage but, beyond periodic articles in the Dogslife newsletter, they are not prompted to do so.
* Where the word Dog* is used, the dog's name is automatically inserted.
