# Supplementary material for: Validity of Internet-Based Longitudinal Study Data: The Elephant in the Virtual Room
Source: J Med Internet Res. 2015 Apr 16;17(4):e96. doi: 10.2196/jmir.3530 (PMC4416133; doi:10.2196/jmir.3530)
Supplement: Supplementary file 2 [file jmir_v17i4e96_app2.pdf]

Appendix 2: Dogslife-specific methodology

The Dogslife questionnaire data was validated by two separate methods. Firstly, randomly selected participants in the project were visited to assess the accuracy of answers given in the first 22 months of the project. The dogs' heights, weights and quantities of food fed were measured and owners opinions were sought on the questionnaire 'ease of use'. Wherever possible the questionnaire uses closed-answer questions but for some questions an additional 'other' option, that generates a free-text box, is required to encompass all the possible answers which could be given. Part of the ease of use assessment involved asking whether available answers were appropriate, so as to limit the number of 'other' responses. All questions subject to validation and their answer options are available in Appendix 1. Revisions made to these questions following the validation process are also included.

The second validation method involved collecting veterinary records from another random sample of the cohort. These records were checked against the online Dogslife records (performed by CP, double-checked by DNC) to determine whether the Dogslife illness and vaccination data for the periods covered by the veterinary records (up to the first 30 months of the project) were consistent with the veterinary records, gave a complete picture of the dogs' health and were sufficient to determine presenting signs and potential diagnoses described in the records.

Participant Visits

Dogs were selected by assigning each a random number using the RAND function in MySQL. The 100 lowest random numbers were chosen from those whose owners had made a data entry in the six months prior to the date of selection (7th March 2012). All were emailed and over 60 provisionally agreed to a visit. Forty-three dogs were visited based on mutually convenient timings. They were distributed geographically across England, Wales and Scotland.

A modified version of the online questionnaire was used for member visits and was intended to serve four purposes:

- Checking basic information, such as household type
- Allowing the visitor to view vaccination cards and measure the dogs
- Ascertaining the ease of use of the online questionnaire
- Facilitating discussion of the Dogslife study protocol

Dog weights were measured using a Nintendo Wii Balance Board (Nintendo Europe: Nintendo Center, PO Box 1501, D-63760, Germany) connected via blue tooth to an Apple MacBook (Apple: 1 Infinite Loop, Cupertino, CA 95014, USA) using the WiiScale i386 application for Mac OS 10.5. In testing of dogs and humans weighing between 5kg and 65kg, it was determined to yield measurements within 0.1kg of an Avery Weigh-Tronix pet scale (ITW Limited, Foundry Lane, Smethwick, West Midlands B66 2LP).

Dog heights were measured using a seca213 stadiometer (SECA UK: Medical Scales & Measuring Systems, 40 Barn Street, Birmingham, B5 5QB) comprising a vertical rule and an attached plate. The dogs were measured to the shoulder by standing them with their

shoulders squarely above their front paws and lowering the sliding plate of the stadiometer to rest on the shoulders.

Collection of Heath Records

When owners register with Dogslife they are asked whether the Dogslife team might contact them via telephone and/or email. Over 95% of Dogslife members have given permission for one or more method, 93% by email and 35% by telephone. One hundred owners were randomly chosen, again using the MySQL RAND function, from those whose owners had indicated that Dogslife could contact them by email or telephone;

- If permitted, telephone contact was attempted to ask whether Dogslife could request their dog's veterinary record.
- If telephone contact failed or telephone contact was not permitted, an email was sent.
- If the owner agreed, a form was sent to them and if they signed and returned it, the vet(s) were contacted once to request the dog's full clinical history.

To increase the number of records obtained, all visited owners were also asked to complete the form. All telephone contact was undertaken by the Dogslife project secretary, who has a strong background in phone-based communications and has developed a rapport with many members since Dogslife's inception.

The veterinary records contained information regarding vaccinations, neuter status, weight, illnesses and partial information regarding worming and flea treatment products. Sixty-six records were collected prior to June 2012 and initial examination suggested that more would be required to understand and quantify the extent of illness under-reporting. On that basis, 142 further veterinary records were sought. The numbers of owners agreeing at each stage of the process are shown in Table A1.

Table A1: Contact Methods and Success Rates

Contact Method	Number Agreed	Signed Forms Returned	Records Obtained	Success Rate
65 Phoned	63	45[2]	45[2]	69%
156 Emailed	61	49[4]	49[4]	31%
21 Uncontactable				
41 Visited	41	41[1]	38[1]	93%
All	165	135[7]	132[7]	46%

Numbers is parentheses [n] indicate extra records from the same owner for second and third Dogslife dogs in the same household.
